# The prognostic value of myeloid derived suppressor cell level in hepatocellular carcinoma: A systematic review and meta-analysis

**DOI:** 10.1371/journal.pone.0225327

**Published:** 2019-12-02

**Authors:** Xinyu Zhang, Xin Fu, Tianyu Li, Huimin Yan

**Affiliations:** 1 Graduate College of Hebei Medical University, Hebei Medical University, Shijiazhuang, Hebei, China; 2 Clinical Research Center, Shijiazhuang Fifth Hospital, Shijiazhuang, Hebei, China; Chang Gung Memorial Hospital at Linkou, TAIWAN

## Abstract

**Background and aims:**

Many studies have investigated the association between the level of myeloid derived suppressor cells (MDSCs) and clinical features and prognosis of hepatocellular carcinoma (HCC), but the results remain controversial. This systematic review and meta-analysis was conducted to summarize all available data and estimate the relationship.

**Methods:**

A comprehensive literature review was carried out using Medline, Embase and Web of Science database through December 2018 to identify relevant studies. The standardized mean difference (SMD) and the hazard ratio (HR) with 95% confidence interval (CI) were utilized for evaluating continuous outcomes and survival analysis, respectively. All statistical analyses were performed by STATA 14.0 software.

**Results:**

A total of 13 studies with 1002 HCC patients were included in the meta-analysis. Overall, the proportion of MDSCs in HCC patients was higher than that in healthy controls (SMD = 4.49, 95% CI = 2.53–6.46, *P*<0.001), and patients with chronic liver disease (SMD = 3.41, 95% CI = 1.58–5.24, *P*<0.001). Subgroup analysis based on the phenotypes of MDSCs and geographical areas showed similar results. However, the frequency of MDSCs was not affected by the treatment with conventional approaches for HCC (SMD = -0.25, 95% CI = -0.57–0.06, *P* = 0.119). Moreover, increased MDSCs level was significantly associated with poorer overall survival (HR = 2.36, 95% CI = 1.70–3.29, *P*<0.001) and recurrence-free survival (HR = 2.72, 95% CI = 1.70–4.35, *P*<0.001), but not significantly correlated with any clinicopathological parameters.

**Conclusion:**

The results of this systematic review suggest that elevated MDSCs level appears to be associated with an increased risk for disease progression and poor prognosis for HCC.

## Introduction

Hepatocellular carcinoma is one of the leading causes of cancer-related death worldwide, which responsible for more than 700,000 deaths each year according to the most recent global cancer statistics in 2018[1]. Despite remarkable improvement in the diagnosis and treatment, HCC remains an intractable disease. The major risk factor of HCC is chronic infection with hepatitis B virus (HBV) or hepatitis C virus (HCV) and subsequent hepatic cirrhosis[2]. The pathogenesis of HCC is not fully understood. Recently, there is a general consensus that various dysfunctions of the immune system contribute to HCC development and progression[3,4]. Therefore, it is important to understand the potential immune modulator in HCC.

Myeloid derived suppressor cells (MDSCs) are a key component of the immunosuppressive network. These cells are capable of impairing innate and adaptive immune responses through various pathways, thereby restricting antitumor immune responses[5,6]. MDSCs have been reported to involve in many carcinomas, such as lung, breast, colorectal and gastric cancers[7–9]. In the last decade, the clinical importance of MDSCs in HCC patients has been investigated, but no consensus has been reached due to multiple phenotypes of MDSCs, the different design, patients population, and so on[10–12]. Furthermore, concerning the prognosis value, MDSCs are expected to be associated with an unfavorable prognosis based on their negative immunoregulated capacity. However, this idea has been challenged by recent studies showing that MDSCs may not be a predictor for the prognosis of overall survival of breast cancer[13]. Therefore, it is desirable to demonstrate whether there is an association between MDSCs and the risk of HCC. In the present meta-analysis, we summarized the results of published studies and evaluated the available evidence to better understand the role played by MDSCs in HCC process.

## Methods

### Search strategy

This meta-analysis was performed according to the PRISMA guidelines. The PRISMA checklist is provided in S1 Table.

A systematical literature search was performed using the Medline, Embase and Web of Science databases to acquire eligible studies published before December 2018. The literature review was independently conducted by two investigators, and the following search terms were used: (“MDSC” OR “MDSCs” OR “Myeloid derived suppressor cell” OR “Myeloid derived suppressor cells”) and (“Hepatocellular carcinoma” OR “HCC” OR “Liver neoplasm” OR “Liver tumor” OR “Liver cancer”). Other potentially eligible studies were also checked through manual searches of reference lists of included studies.

### Inclusion and exclusion criteria

The inclusion criteria were as following: studies that evaluated the level of MDSCs and/or the association between MDSCs level and HCC risk; peripheral MDSC are defined as “HLA-DR^low/−^CD11b^+^CD33^+^” or “HLA-DR^low/-^CD14^+^”; the HCC patients were clearly diagnosed; providing sufficient published data for the meta-analysis; studies were written in English. The exclusion criteria were as follows: reviews, conference abstracts, case reports, and letters; articles about cell lines or animals.

### Data extraction and quality assessment

Two investigators independently extracted the data from all available studies. The discrepancies in article information were resolved via discussion and consensus.

The following information was collected from each study: first author, publication year, country, sample source, the number of participants, therapeutic method, measure method of MDSCs, markers to identify MDSCs, frequency of MDSCs, Hazard ratio (HR) with 95% confidence interval (CI), and prognostic outcome. If the HRs and 95% CIs were not reported directly in the papers, they were estimated from Kaplan-Meier curves according to the methods reported by Tierney et al.[14].The Newcastle-Ottawa Quality Assessment Scale for cross-sectional study was used to assess the quality of the included studies[15]. The scores ranged from 0 to 9, with a score ≥ 6 indicating good quality.

### Statistical analysis

Standardized mean difference (SMD) and 95% CI were employed to compare the difference in MDSCs level between HCC patients and controls, or before and after therapy. Subgroup analysis was performed according to the phenotypes of MDSCs and geographical areas. HR combined with 95% CI were calculated to evaluate the association between MDSCs level and prognosis of HCC patients. Heterogeneity among the included studies was evaluated using Chi-square based Q test and *I*^*2*^-statistic test. A *P* value <0.05 was considered significant. If heterogeneity existed, a random effect model was used. Otherwise, a fixed-effect model was applied. Publication bias was assessed using Begg's funnel plot and Egger's test. All statistical analyses were performed by STATA 14.0 software.

## Results

### Study selection

**Fig 1:**
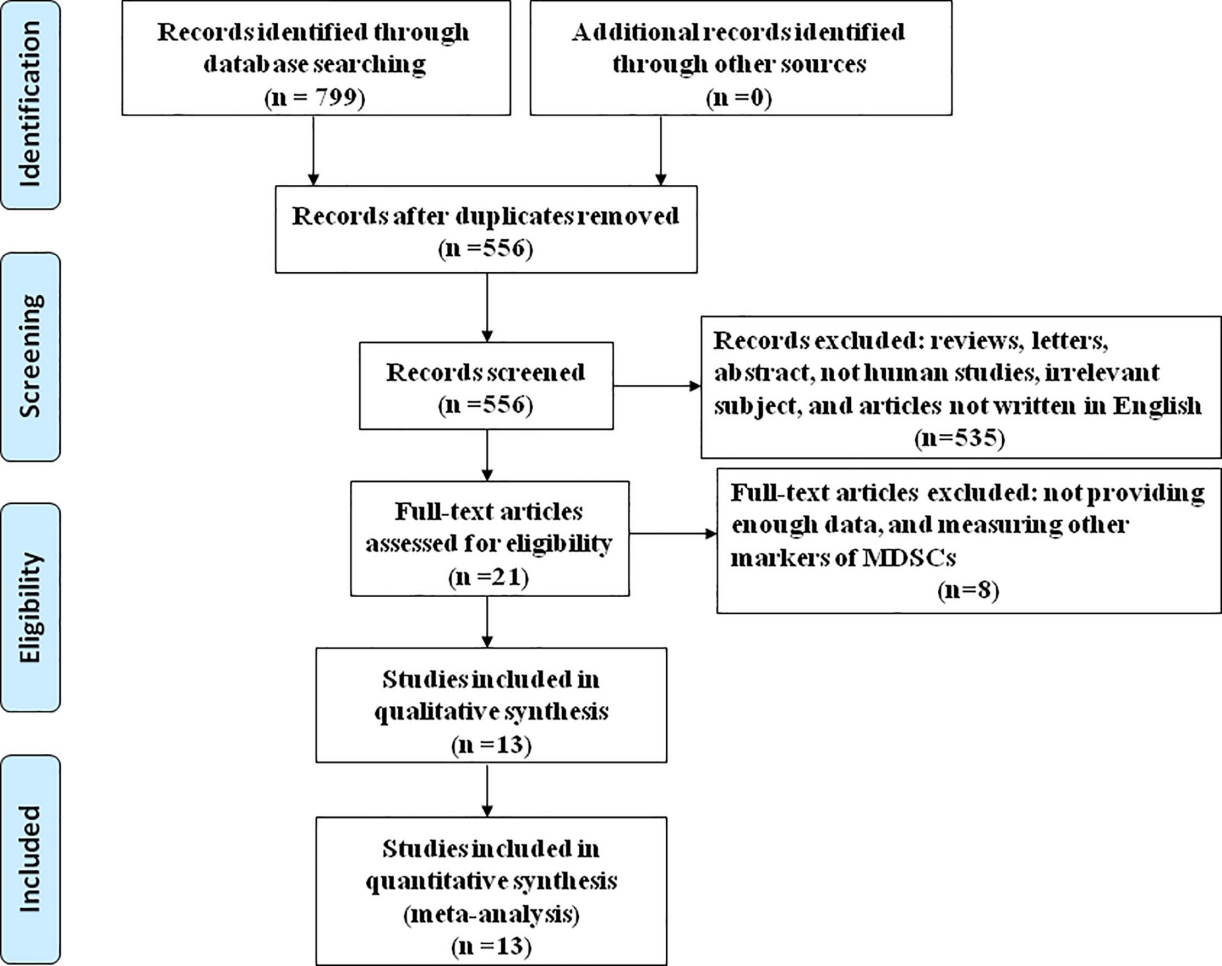
Flowchart summarizing literature search strategy and selection of studies.

A flowchart of the study selection process was shown in Fig 1. After searching the databases, we obtained 556 potentially relevant articles without duplication. In a primary screening of titles and abstracts, 535 studies were excluded. After further evaluation of the full text, 8 articles were excluded. Finally, a total of 13 publications with 1002 HCC patients were used to perform the meta-analysis.

### Characteristics of the included studies

The main characteristics of the 13 studies[10,16–27] were summarized in Table 1 and S2 Table. All of the eligible studies were published by groups in USA, China, Japan, and Egypt between 2008 and 2018. Nine studies assessed the level of MDSCs in HCC patients and healthy controls, and 6 studies evaluated the level of MDSCs in HCC patients and patients with chronic liver disease. Additionally, 6 articles compared the level of MDSCs in patients before and after therapy, which included sorafenib, chemotherapy, radiofrequency ablation (RFA), and transarterial chemoembolization (TACE). As for the survival outcomes, 5 studies had data for overall survival (OS), 3 studies for disease-free survival (DFS)/time-to-recurrence (TTR). With regard to the determination of MDSCs, FACS was used in 11 studies, while 2 studies used quantitative real-time PCR and immunohistochemistry.

**Table 1:** The baseline characteristics of included studies.

First author, year	Country	Sample source	No. of HCC	Treatment	MDSC definition	Measure method	Cut-off	Outcome
Elwan 2018	Egypt	PB	20	NR	Lin^-^HLA-DR^-^CD11b^+^CD33^+^	FACS	NR	NR
Zhou, 2018	China	PB, Tumor tissue	PB:26 Tissue:102	NR	PB:HLA-DR^-^CD11b^+^CD33^+^ Tissue:CD11b/CD33/CCRK	PB:FACS Tissue:qRT-PCR	Median	OS, RFS
Li, 2017	China	PB	55	NR	HLA-DR^-/low^CD11b^+^CD33^+^	FACS	NR	NR
Deng, 2017	China	Tumor tissue	78	NR	CD11b^+^	IHC	Median	OS
Gao, 2017	China	PB	183	Surgery	HLA-DR^-/low^CD14^+^	FACS	Median	OS,TTR
Iwata, 2016	Japan	PB	122	RFA and TACE	PDL1^+^HLA-DR^-/low^CD11b^+^CD33^+^CD14^+^	FACS	Median	NR
Kalathil, 2016	USA	PB	19	Sorafenib	CD11b^+^CD33^+^	FACS	NR	NR
Mizukoshi, 2016	Japan	PB	36	Chemotherapy	HLA-DR^-/low^CD14^+^	FACS	Median	NR
Wang, 2016	China	PB	92	Radiotherapy	HLA-DR^-/low^CD14^+^	FACS	Average+2SD	OS
Arihara, 2013	Japan	PB	123	NR	HLA-DR^-/low^CD14^+^	FACS	Average+2SD	OS, RFS
Kalathil, 2013	USA	PB	23	NR	HLA-DR^-^CD11b^+^CD33^+^CD14^-^	FACS	NR	NR
Mizukoshi, 2013	Japan	PB	12	RFA	HLA-DR^-/low^CD14^+^	FACS	NR	NR
Hoechst, 2008	Germany	PB	111	NR	HLA-DR^-/low^CD14^+^	FACS	NR	NR

*Abbreviations*: NR, not reported; PB, peripheral blood; IHC, immunohistochemistry; RFA, radiofrequency ablation; TACE, trans arterial chemo-embolization; FACS, fluorescence-activated cell sorting; OS, overall survival; RFS, recurrence-free survival; TTR, Time to recurrence

### Proportion of peripheral MDSCs in HCC patients

**Fig 2:**
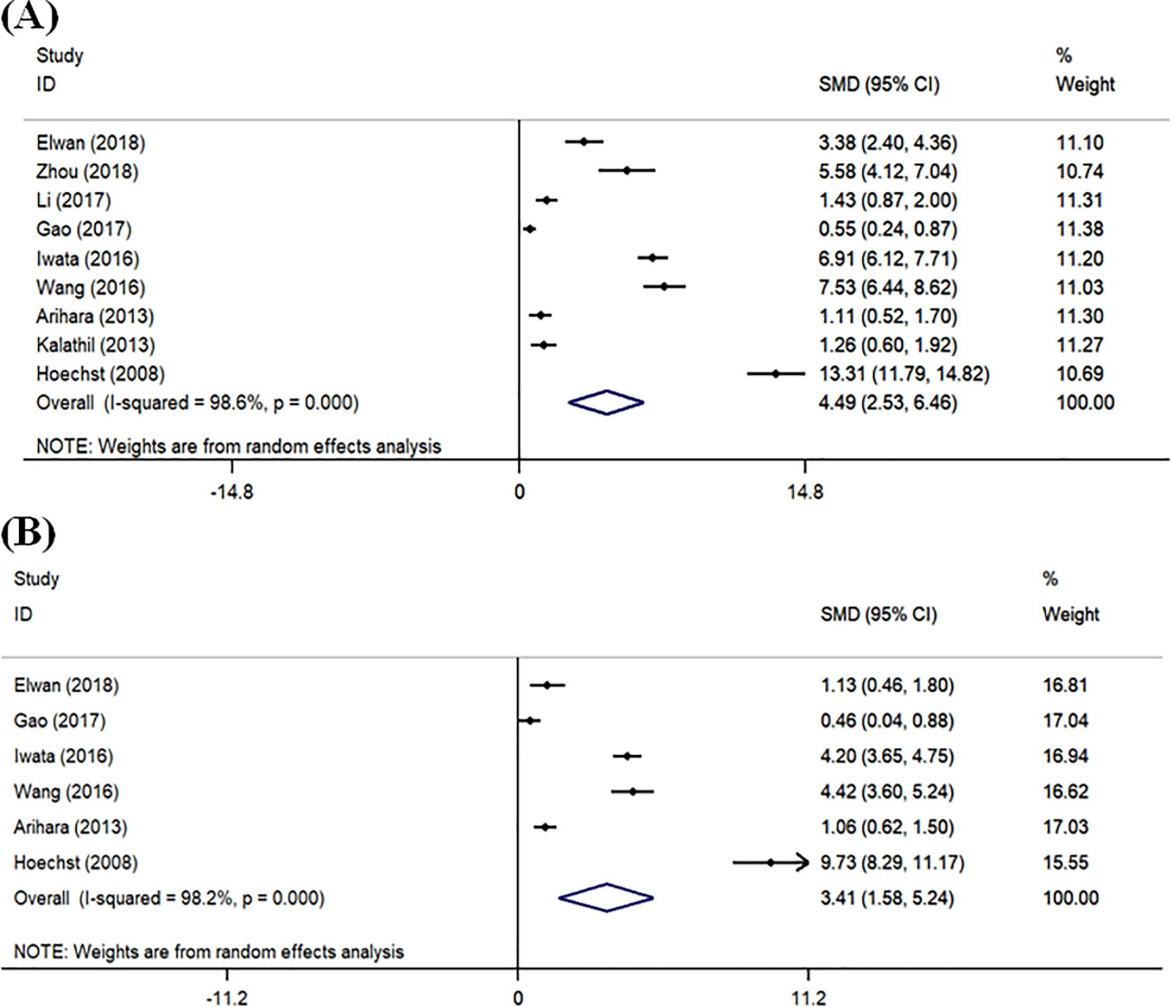
Forest plot of meta-analysis evaluating the proportion of MDSCs in HCC patients and healthy controls (A) or HCC patients and patients with chronic liver disease (B).

As shown in Fig 2, the pooled analysis showed that the proportion of peripheral MDSCs in HCC patients was higher than that in healthy controls (SMD = 4.49, 95% CI = 2.53–6.46, *P*<0.001), and patients with chronic liver disease (SMD = 3.41, 95% CI = 1.58–5.24, *P*<0.001). Heterogeneity assessed by *I*^2^ statics was 98.6% (*P*<0.001) and 98.2% (*P*<0.001), respectively.

**Fig 3:**
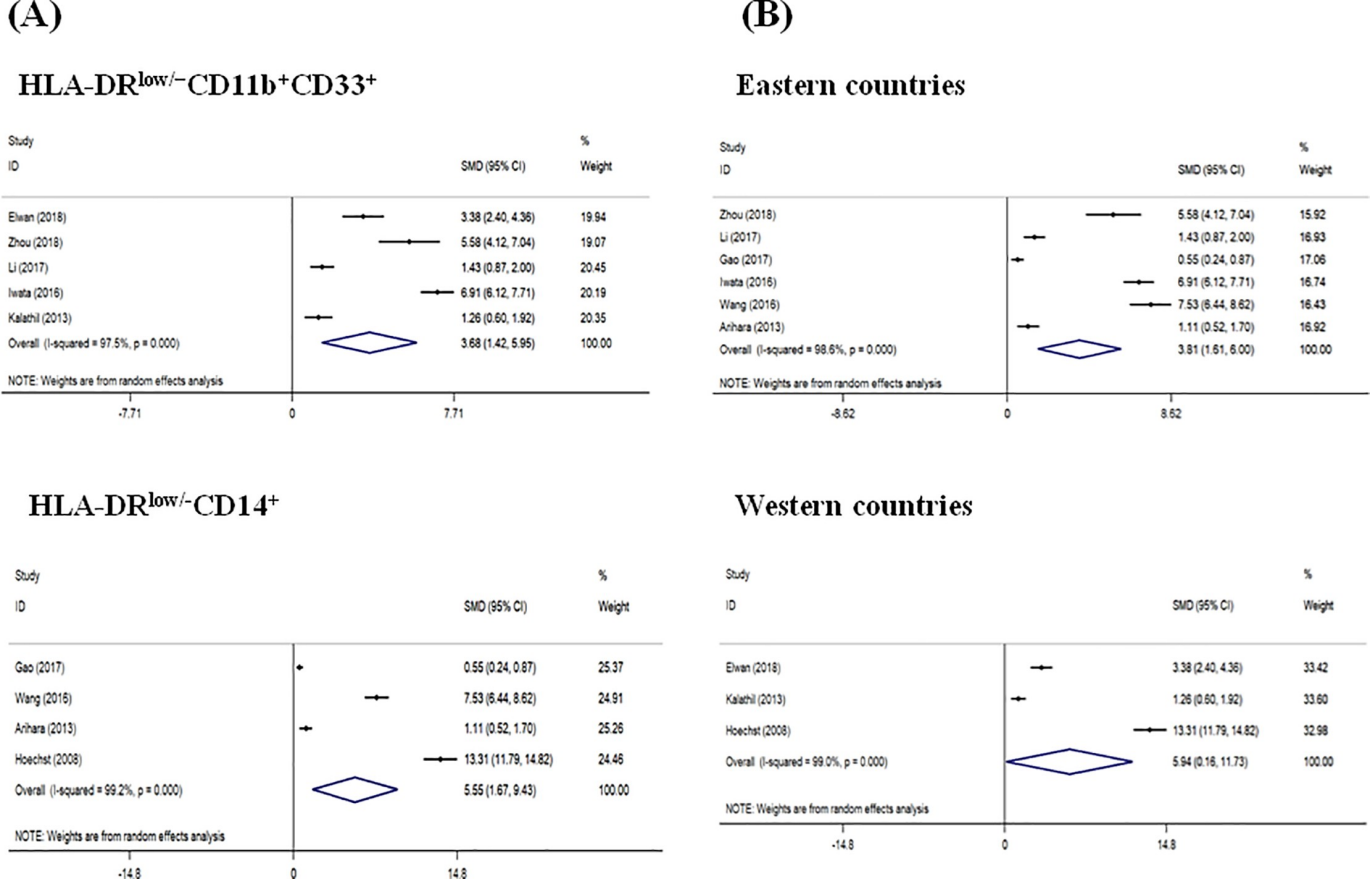
Forest plot of subgroup meta-analysis evaluating the proportion of MDSCs by the phenotypes of MDSCs (A) and geographical areas (B) in HCC patients and healthy controls.

Subgroup analysis was performed according to the phenotypes of MDSCs (Fig 3A). Pooled data of 5 studies in which MDSCs were identified as “HLA-DR^low/−^CD11b^+^CD33^+^” revealed that there was a significantly higher frequency of MDSCs in HCC patients compared to healthy controls (SMD = 3.68, 95% CI = 1.42–5.95, *P* = 0.001). There was also a significant increase in the frequency of MDSCs in HCC patients compared to healthy controls when MDSCs were identified as “HLA-DR^low/-^CD14^+^” (SMD = 5.55, 95% CI = 1.67–9.43, *P* = 0.005). For subgroup analysis of the geographical areas, 6 studies were performed in Eastern countries, and the remaining 3 in Western countries (Fig 3B). The pooled analysis showed that the frequency of MDSCs was significantly higher in HCC patients compared with healthy controls in both Eastern (SMD = 3.81, 95% CI = 1.61–6.00, *P* = 0.001) and Western (SMD = 5.94, 95% CI = 0.16–11.73, *P* = 0.044) countries.

### Effect of treatment on MDSCs in HCC patients

**Fig 4:**
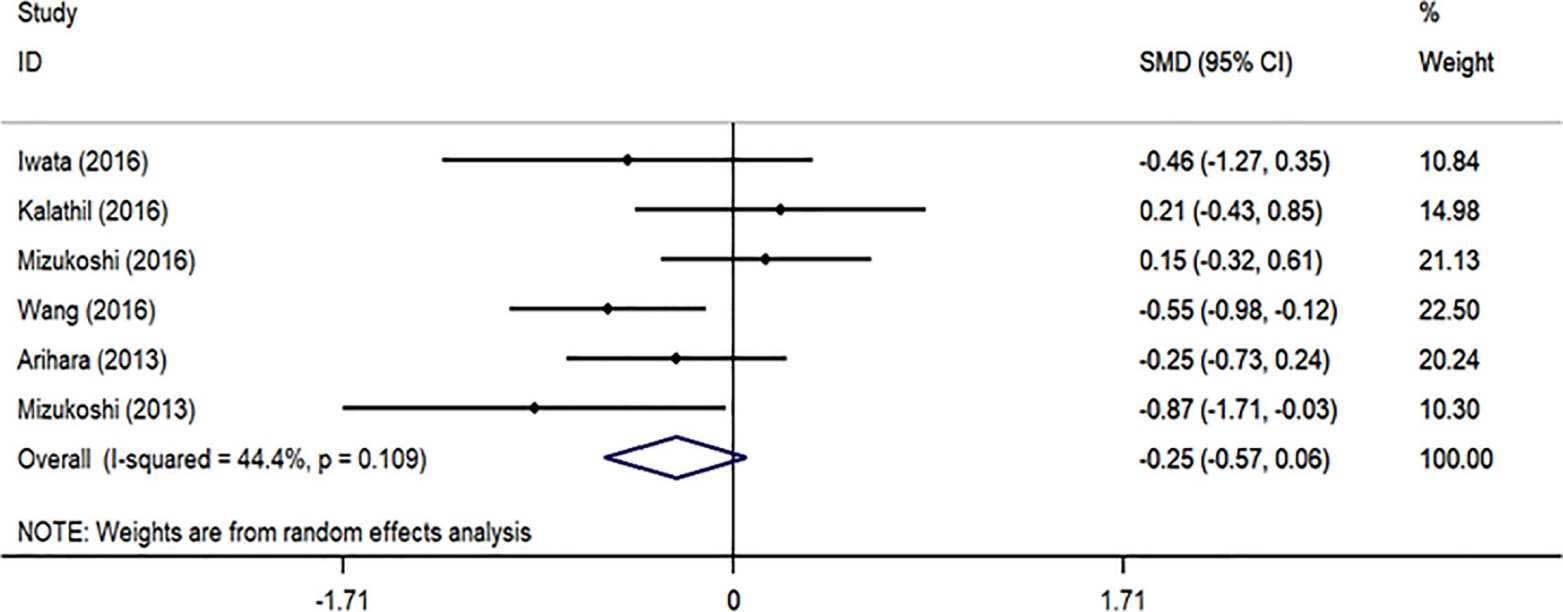
Forest plot of meta-analysis evaluating the proportion of MDSCs before and after therapy in HCC patients.

To further assess the effect of treatments on MDSCs level, we analyzed 6 studies that compared the proportion of MDSCs before and after therapy in HCC patients (Fig 4). There was no significant difference between two groups (SMD = -0.25, 95% CI = -0.57–0.06, *P* = 0.119). Heterogeneity assessed by *I*^*2*^ statics was 44.4% (*P* = 0.109).

### Association between MDSCs and survival of HCC patients

**Fig 5:**
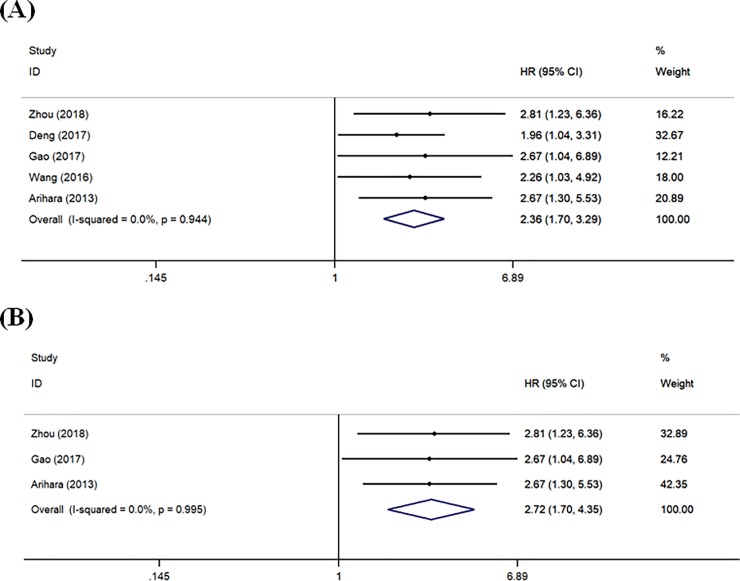
Forest plot of meta-analysis evaluating the relationship between MDSCs level and OS (A) or RFS/TTR (B) in HCC patients.

There were 5 studies reporting HRs for OS (Fig 5A). Overall, higher MDSCs level was significantly associated with poorer OS (HR = 2.36, 95% CI = 1.70–3.29, *P*<0.001). Heterogeneity assessed by *I*^2^ statics was 0% (*P* = 0.944). In addition, stratified analyses were carried out according to the specimen source. In these 5 studies, 2 used liver tissue samples and 3 used peripheral blood samples. High expression of both circulating (HR = 2.52, 95% CI = 1.58–4.00, *P*<0.001) and tissue-based (HR = 2.21, 95% CI = 1.38–3.55, *P* = 0.001) MDSCs was significantly associated with unfavorable OS of HCC patients (Data not shown).

There were 3 studies reporting HRs for RFS/TTR (Fig 5B). The pooled estimate showed that HCC patients with higher MDSCs had shorter RFS/TTR (HR = 2.72, 95% CI = 1.70–4.35, *P*<0.001). Heterogeneity assessed by *I*^2^ statics was 0% (*P* = 0.995).

### Correlation of MDSCs level with clinicopathological features

As shown in Table 2, MDSCs level was not significantly correlated with any clinicopathological parameters evaluated in this study, including gender, virological markers, tumor size, liver cirrhosis, number of tumors, tumor differentiation, TNM stage, vascular invasion, and lymph node metastasis.

**Table 2:** Main results for meta-analysis between MDSC and clinicopathological factors.

Characteristics	No. of studies	No. of patients	Pooled OR (95%CI)	*P* value
Gender (male *vs*. female)	3	398	1.620 (0.980–2.678)	0.060
HBsAg/HBV/HCV (positive *vs*. negative)	2	275	2.009 (0.951–4.245)	0.067
Tumor size (cm) (>5 *vs*. ≤5)	1	183	0.651 (0.351–1.211)	0.175
Liver cirrhosis (yes *vs*. no)	1	183	0.888 (0.473–1.666)	0.712
Number of tumors (multiple vs solitary)	3	398	2.067 (0.785–5.441)	0.141
Tumor differentiation (poor/moderate *vs*. well)	1	183	0.677 (0.375–1.225)	0.197
TNM stage (III/IV) *vs*. I/II)	2	215	1.349 (0.073–24.994)	0.841
Child-Pugh score (B+C) *vs*. (0+A)	3	398	0.823 (0.139–4.869)	0.830
Vascular invasion (yes *vs*. no)	1	183	2.180 (1.154–4.118)	0.016

### Publication bias

**Fig 6:**
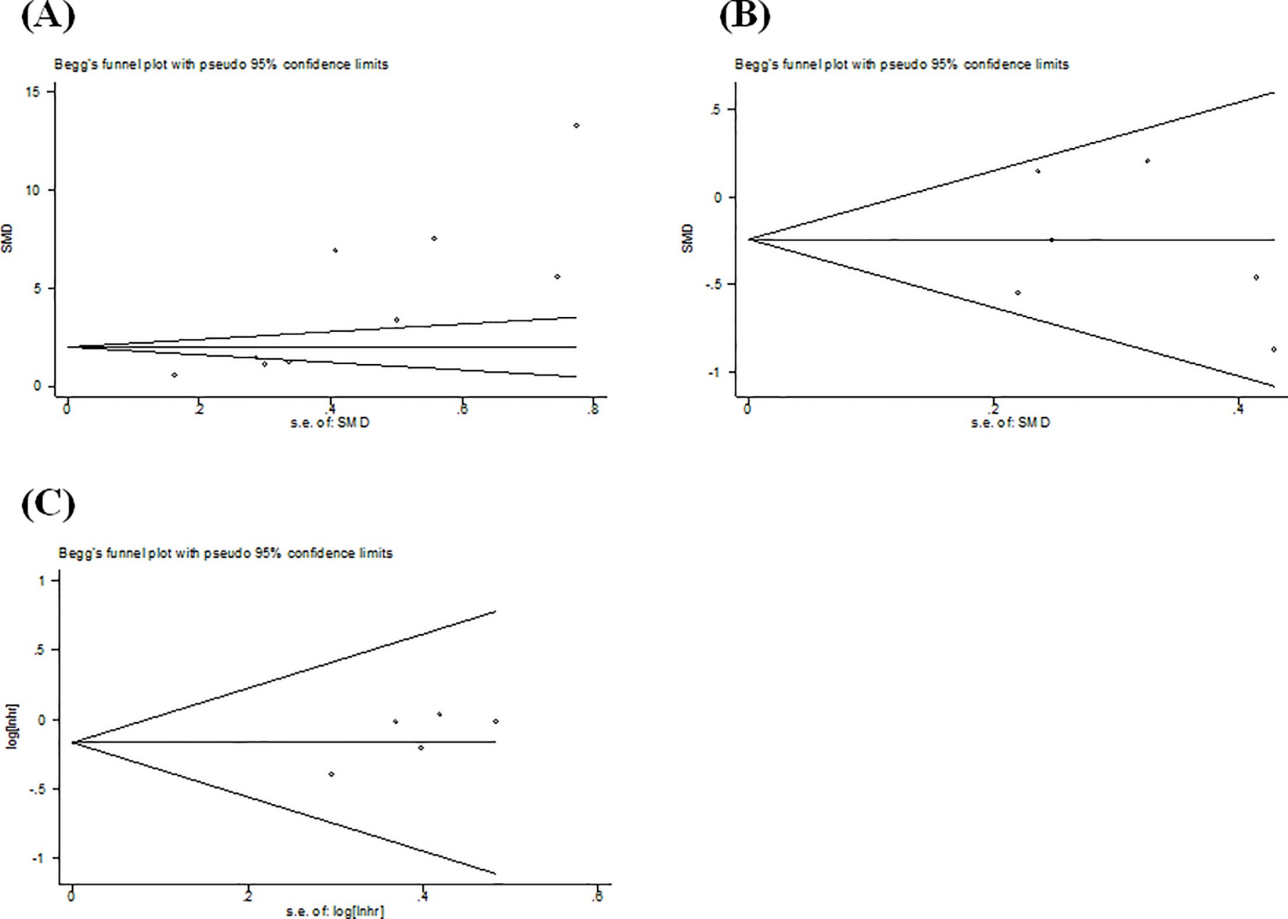
Funnel plot of publication bias for the studies evaluating MDSCs proportion in HCC patients and healthy controls. (A), treatment effect (B), and overall survival (C).

Begg’s test was performed to estimate the publication bias. As shown in Fig 6, there was evidence of publication bias for studies evaluating the proportion of MDSCs in HCC patients and healthy controls (*P*<0.001). The Trim and Fill method was applied to address this problem, and showed a pooled adjusted SMD of 1.02 (95% CI = 0.82–1.21, *P*<0.001), which remained statistically significant. There were no significant publication biases for studies included in the analysis of treatment effect (*P* = 0.669) and OS analysis (*P* = 0.091).

## Discussion

As a novel immunosuppressive cell population, MDSCs appear to facilitate tumor immune escape by inhibiting antitumor immune response[5,6,28]. Recently, more and more studies focus on the clinical role of MDSCs in HCC, but the results are controversial. The present meta-analysis summarized all relevant studies, and evaluated the proportion of MDSCs in HCC patients. Moreover, the clinicopathological and prognostic value of MDSCs for HCC was also assessed.

We firstly performed a meta-analysis of studies measuring the frequency of MDSCs in HCC patients. To our knowledge, this is the first meta-analysis to elucidate MDSCs status in such patients. Pooled data revealed that the percentage of MDSCs in HCC patients was significantly higher than that in patients with chronic liver disease and healthy controls. We also performed a meta-analysis to compare the percentage of MDSCs in patients with chronic liver disease and healthy controls, and found that patients with chronic liver disease had a significantly higher percentage of MDSCs (SMD = 2.71, 95% CI = 0.89–4.53) (Data not shown). These results suggested that increased MDSCs may contribute to the progression from chronic hepatitis to HCC.

To address the clinical heterogeneity among the studies, subgroup analyses were done according to the geographical areas. The results indicated that the association remained statistically significant. We also performed a subgroup analysis based on the markers for MDSCs. The results revealed that HCC patients had a higher proportion of MDSCs compared to healthy controls, regardless of whether MDSCs were defined as “HLA-DR^low/−^CD11b^+^CD33^+^” or “HLA-DR^low/-^CD14^+^”, two most commonly used markers of MDSCs in HCC patients. It seems that the different phenotypic markers of MDSCs do not affect the conclusion. Over the last years, there is a change in the definition of MDSCs[29,30]. At present, HLA-DR^low/−^CD11b^+^CD33^+^ cells are considered to be total MDSCs, whereas HLA-DR^low/-^CD14^+^ cells are used to identify monocytic MDSCs [31,32]. Therefore, more studies with larger sample sizes are needed to value the real status of MDSCs in patients with HCC. It is unclear the effect of HCC treatment on MDSCs level. Several studies have investigated the change of MDSCs frequency in patients receiving curative therapy, such as chemotherapy, surgical resection, sorafenib, RFA and TACE[17–20,22,23]. We summarized the results of these studies, and found that there was no significant difference in the proportion of MDSCs between before and after treatment. This finding suggested that there was only a limited effect of current clinical therapeutic options on MDSCs. Therefore, the combination of routine treatment and inhibition of MDSCs could be an effective strategy in improving clinical outcome for HCC patients. However, subgroup analysis based on every therapy could not be conducted due to the limitation of sample size. Considering that the different therapeutic approaches might have different effects on MDSCs, more clinical studies are needed to verify the above conclusion.

Abnormally increased MDSCs might contribute to poor prognosis of cancer patients. Several previous systematic reviews have evaluated the prognostic value of MDSCs in all types of solid tumors[33–35]. In these studies, the relationship between MDSCs and prognosis of HCC was also evaluated, but the number of relevant studies was very small. Different from the above studies, the present study collected more recent data and provided more comprehensive information. Our pooled data revealed that higher MDSCs level significantly correlated with short OS and RFS. We carried out the stratified analysis based on the specimen source, and found that the HRs for OS were similar between the subgroups. The results provide support that the change of circulating and tissue-based MDSCs level could be utilized as an independent factor in predicting the prognosis of HCC patients. As for the clinicopathological features, our analysis revealed that MDSCs expression did not correlate with any clinicopathological variables. This is the first meta-analysis to systematically explore the relationship between MDSCs level and clinicopathological factors of HCC patients. However, this conclusion needs to be further verified because of the small number of studies.

There were some limitations in our meta-analysis. First, the cutoff values defining high MDSCs expression were different across studies. These may contribute to the heterogeneity in our meta-analysis. Second, some studies did not provide complete data, therefore we used the extracted data. This could influence the accuracy of data. Third, the number of studies related to clinical treatment was relatively insufficient. Hence, no stratified analysis was performed. Finally, studies with positive data are more likely to be published, which may lead to overestimated correlation. Therefore, more researches are needed to verify the results obtained by the current study.

## Conclusion

In conclusion, the results of this meta-analysis suggest that there is a significant association between higher MDSCs level and an increased risk and poor survival of HCC. The status of MDSCs could be important for maintaining tumor microenvironment to facilitate tumor growth. MDSCs might be utilized as a prognostic marker for HCC. Considering the phenotypical and functional heterogeneity of MDSCs, more clinical studies are necessary to verify our conclusion.

## Supporting information

S1 Table PRISMA checklist. (DOC)

S2 Table Additional characteristics of included studies. (DOCX)
