# Supplementary material for: The prognostic value of myeloid derived suppressor cell level in hepatocellular carcinoma: A systematic review and meta-analysis
Source: PLoS One. 2019 Dec 2;14(12):e0225327. doi: 10.1371/journal.pone.0225327 (PMC6886785; doi:10.1371/journal.pone.0225327)
Supplement: S2 Table — (DOCX) [file pone.0225327.s002.docx]

**S2 Table Additional characteristics of included studies**

| First author, year | HCC patiens | | | |  | Quality assessment | | |
| --- | --- | --- | --- | --- | --- | --- | --- | --- |
|  | No. of patients | Age | Gender  (M/F) | Etiology |  | Selection | Comparability | Outcome |
| Elwan 2018 | 20 | 54.1±7.1 | 11/9 | NR |  | ★★ | ★★ | ★★ |
| Zhou, 2018 | 102 | NR | 82/20 | HBV/HCV/others |  | ★★★ | ★ | ★★★ |
| Li, 2017 | 55 | 56.4 (32.0-79.0) | 52/3 | NR |  | ★★★ | ★ | ★★ |
| Deng, 2017 | 78 | NR | NR | NR |  | ★★★ | ★ | ★★★ |
| Gao, 2017 | 183 | 56 (24-81) | 152/31 | NR |  | ★★★★ | ★★ | ★★★ |
| Iwata, 2016 | 122 | 63.8±9.4 | 83/39 | HBV/HCV/others |  | ★★★★ | ★★ | ★★ |
| Kalathil, 2016 | 19 | 62 (45-83) | 16/3 | HBV/HCV/others |  | ★★ | ★★ | ★★ |
| Mizukoshi, 2016 | 36 | 62.7±8.5 | 32/4 | NR |  | ★★★★ | ★★ | ★★ |
| Wang, 2016 | 92 | 54.0±10.4 | 72/20 | NR |  | ★★★★ | ★★ | ★★★ |
| Arihara, 2013 | 123 | NR | 83/40 | HBV/HCV/others |  | ★★★★ | ★★ | ★★★ |
| Kalathil, 2013 | 23 | 64 (40-82) | 16/7 | HBV/HCV/others |  | ★★★ | ★ | ★★ |
| Mizukoshi, 2013 | 12 | NR | NR | HBV/HCV/others |  | ★★ | ★★ | ★★ |
| Hoechst, 2008 | 111 | 64 | 91/20 | HBV/HCV/others |  | ★★★★ | ★★ | ★★ |
